# Melanotic neuroectodermal tumor of infancy successfully treated with metformin: A case report

**DOI:** 10.1097/MD.0000000000022303

**Published:** 2020-11-06

**Authors:** Yu Liang, Ruicheng Tian, Jing Wang, Yuhua Shan, Hongxiang Gao, Chenjie Xie, Jingjing Li, Lei Zhang, Min Xu, Song Gu

**Affiliations:** Department of Surgery, Shanghai Children's Medical Center, Shanghai Jiaotong University School of Medicine, Shanghai, China.

**Keywords:** case report, melanotic neuroectodermal tumor of infancy, metformin, novel therapeutics

## Abstract

**Rationale:**

Melanotic neuroectodermal tumor of infancy (MNTI) is a rare tumor originated from neural crest cells with the potential for recurrence and metastasis. The peak age for the disease is during the first year after birth. The current therapy is primarily surgery. The patient reported here is the first case of MNTI treated with metformin.

**Patient concerns:**

A case of a 4-month-old infant with a history of swelling in the mouth for 1 month.

**Diagnosis:**

The tumor was diagnosed using radiology, pathology, and immunohistochemistry, and it was performed with complete surgical resection. Unfortunately, the tumor recurred 3 months after surgery.

**Interventions:**

We prescribed metformin for the infant.

**Outcomes:**

Currently, after 9 months of treatment, the tumor is well controlled without apparent side effects.

**Lessons:**

The case presented suggested that metformin may be an underlying therapy for MNTI.

## 1 Introduction

MNTI is considered to be a rare tumor throughout the world. Since Krompecher first discovered the tumor in 1918,^[1]^ approximately 500 cases have been reported worldwide. In the past, because the origin of the neoplastic tissue was unknown, it was named “congenital melanoma,” “retinal basal tumor,” “mutant melanoma tumor,” and others.^[2]^ In 1966, Borello and Gorlin^[3]^ discovered that the level of vanillic acid (VMA) in the urine of a 3-month-old infant with MNTI was increased, and returned to normal following surgery. This observation suggested that it was likely that the tumor was of neural crest cell origin, which was subsequently confirmed.^[4–6]^ To date, approximately 500 cases of MNTI have been reported worldwide. Among them, most cases were observed in infants less than 1 year of age, and more commonly in males. MNTI predominantly involves the maxilla, which accounts for about 60% of the cases. Although MNTI is typically a benign lesion that usually is derived from neural crest cells, it has a high risk of recurrence, and in approximately 6.5% of patients, peripheral lymph nodes are involved, or metastasis to distant organs occurs.^[7,8]^

We present a case of an infant with NMTI for whom the tumor recurred after resection and was subsequently treated with metformin. After 9 months of treatment, the tumor had not progressed, and no metastasis was observed. Follow-up examinations are continuing. Metformin, which is commonly used to treat type II diabetes mellitus, has not been reported as a treatment for NMTI previously. Therefore, we provided this detailed case report.

## 2 Case presentation

**Figure 1:**
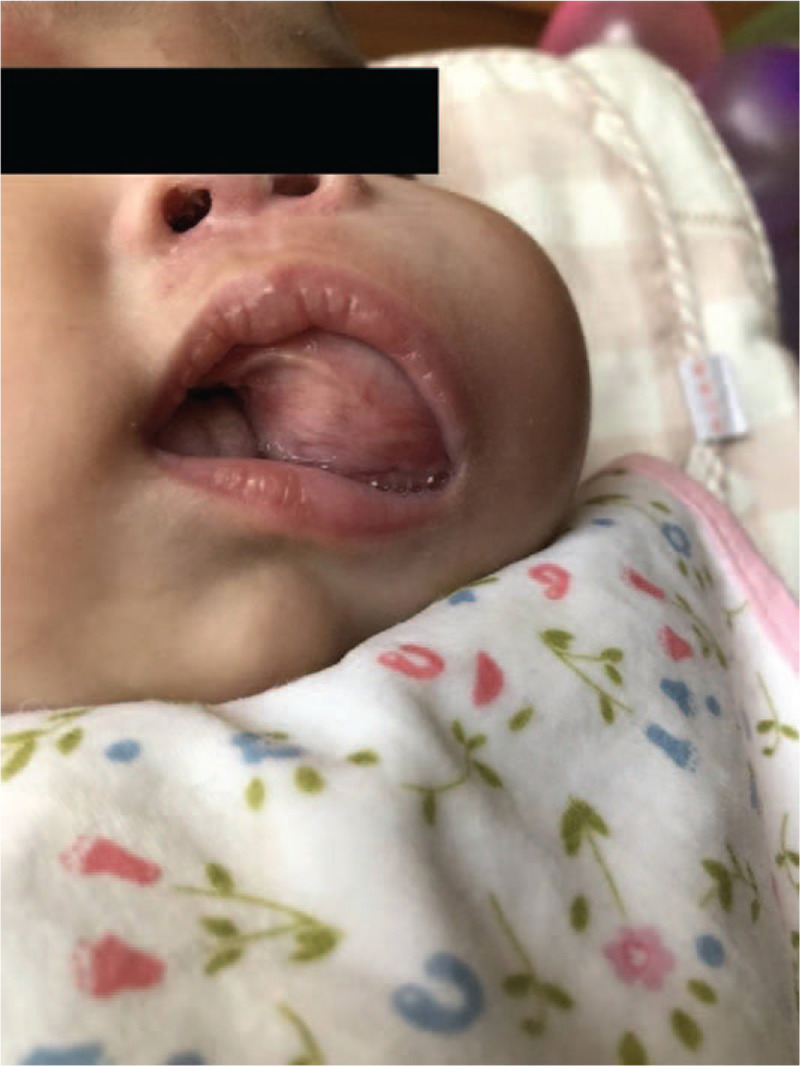
The babys left face was slightly swollen, and a mass 5 cm × 4 cm × 4 cm in size filled the left side of the oral cavity, which made it difficult for the infant to close her mouth.

A 4-month-old baby girl was admitted into our hospital with a mass on the left side of her mouth that had been detected for over a month. One month before her presentation at the hospital, the infants parents noticed a mass approximately 5 cm × 4 cm × 4 cm in size on the left side of her mouth. A local hospital provided a diagnosis of mumps, which turned out to be incorrect. The mass continued to gradually increase in size over time. Therefore, the parents came to our hospital for additional diagnosis and treatment. A slight swelling was visible on the left side of the infants face, and a mass of 5 cm × 4 cm × 4 cm in size filled the left side of the oral cavity. It was difficult for the infant to close her mouth (Fig. 1).

**Figure 2:**
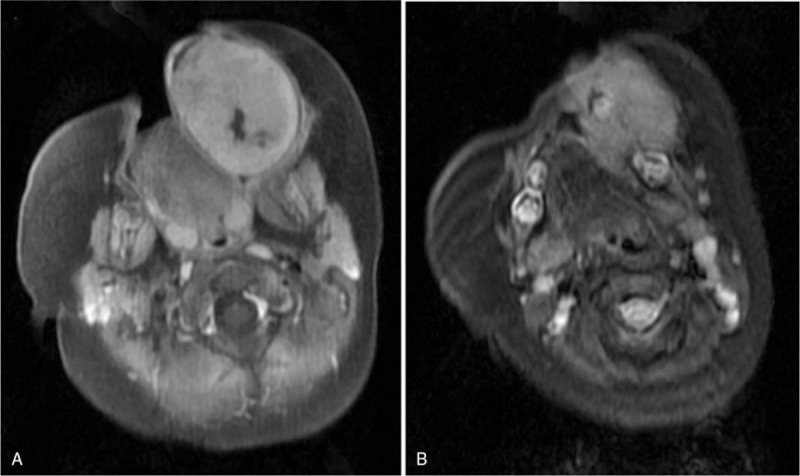
The MRI indicated the presence of a large mass of abnormal signal in the left oral and maxillofacial region that expanded into the peripheral area. The mass was noticeably different from the peripheral tissue on enhanced MRI. The size of the mass was 54 mm × 39 mm × 53 mm. The left maxillary, alveolar bone, and maxillary sinus wall were partly involved.

**Figure 3:**
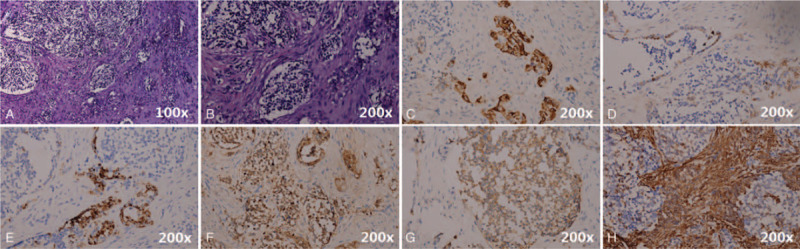
Hematoxylin-eosin [HE] neuroblast-like clusters of small round cells are present in the fibrous tissue with melanin pigmentation. Immunohistochemical staining revealed positive expression for(C) cytokeratin, (D) EMA, (E) HMB-45, (F) neuron-specific enolase, (G) SYN, and (H) VIM. Glial fibrillary acidic protein (GFAP), S100, CD99, DES, LCA, SOX10, SMA, TDT, and CD34 were negative (data not shown).

**Figure 4:**
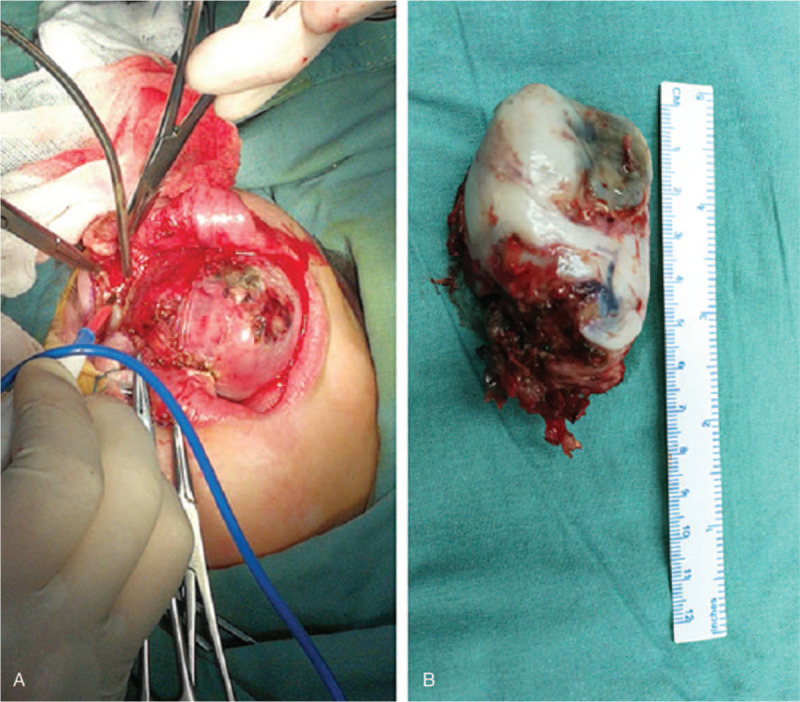
(A) Complete resection of the tumor using a 2 mm margin around the tumor. (B) The maximum diameter of the tumor was nearly 7 cm.

**Figure 5:**
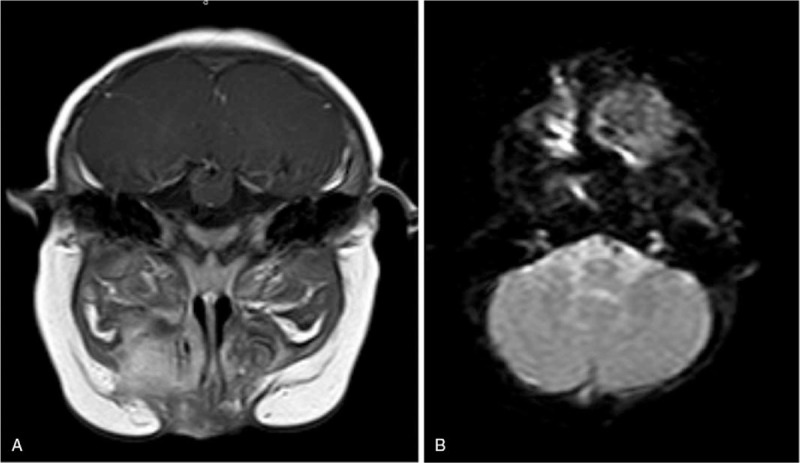
The nasopharynx MRI showed the presence of a new mass of abnormal signal in the same region as before, with a size of 28.8 mm × 28.3 mm × 11.6 mm.

Based on the patients history, the infant was normal at birth and no pregnancy or delivery complications were noted. On physical examination, the infant appeared well-fed and had no apparent history of medication or surgery, nor did she have any known allergies. After admission (2018.12.28), a head MRI scan was performed. The MRI indicated the presence of a large mass with abnormal signal in the left oral and maxillofacial region, which expanded into the peripheral area. The T1WI image revealed a low signal, the T2WI showed a high signal, and the diffusion of DWI was limited. It was observed that the mass was noticeably different from the peripheral tissue on enhanced MRI, and the size of the mass was 54 mm × 39 mm × 53 mm. The left maxillary, alveolar bone, and maxillary sinus wall were partly involved (Fig. 2). Histopathology revealed the presence of clusters of small round cells in the fibrous tissue that were melanin pigmented. Immunohistochemical staining demonstrated that the cells were positive for cytokeratin (CK) EMA, HMB-45, neuron-specific enolase (NSE), SYN, and VIM (Fig. 3). The cells were negative for glial fibrillary acidic protein (GFAP), S100, CD99, DES, LCA, SOX10, SMA, TDT, and CD34 (Fig. 3). Combined morphology and immunohistochemistry confirmed that the tumor was a “melanotic neuroectodermal tumor of infancy”. Therefore, complete resection of the tumor was performed under general anesthesia, with a 2 mm margin around the tumor (Fig. 4). Unfortunately, 3 months later (2019.4.9), the patient was readmitted to our hospital, and the MRI of the nasopharynx demonstrated the presence of a new mass of abnormal signal in the same region as before. The size of the mass was 28.8 mm × 28.3 mm × 11.6 mm. It was clear that the tumor had recurred (Fig. 5).

Considering the young age of the patient and excessive trauma of a second surgery, we suggested metformin (Sino-American Shanghai Squibb Pharmaceuticals Ltd, 0.5 g/ tablet) as a treatment for the tumor. We prescribed an oral dose of 7 mg/kg to be given 3 times a day after meals.

**Figure 6:**
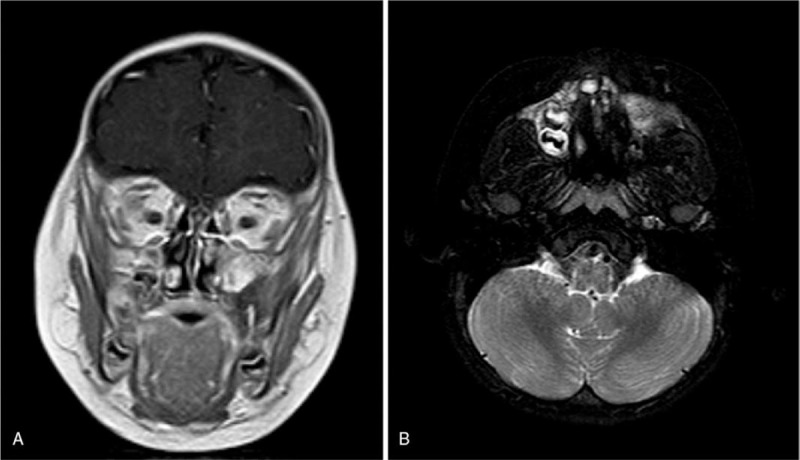
After 9 months of metformin treatment, the nasopharynx MRI (2020.1.8) showed that the size of the tumor was unchanged and was the same as seen on April 9, 2019.

**Figure 7:**
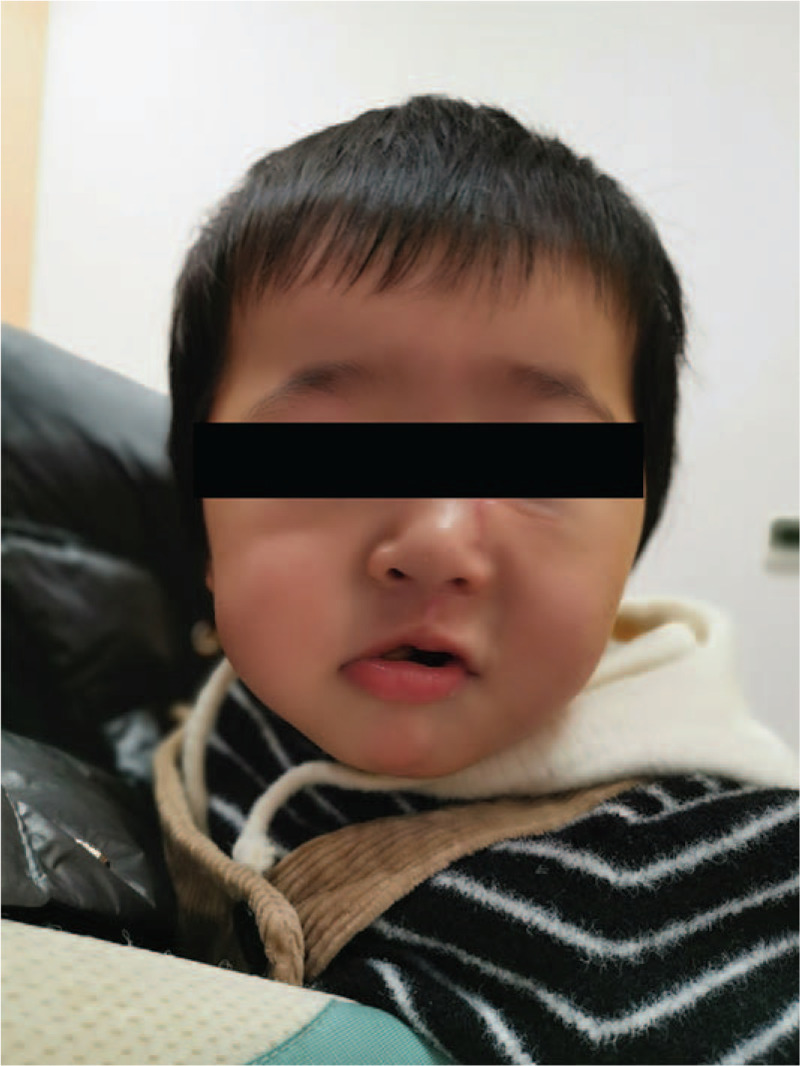
The babys incision healed well, and she exhibited growth and development similar to her peers. No other adverse reactions were noted for the infant.

At the 9-month follow-up examination, the parents reported no remarkable discomfort in the infant due to the medication. The MRI of the nasopharynx region (2020.1.8) revealed that the size of the tumor was unchanged (Fig. 6). Our observations indicated that the tumor was well controlled, and the growth and appetite of the infant were normal (Fig. 7). The parents were recommended to continue oral metformin treatment for the patient and to have regular follow-up examinations.

## 3 Discussion

MNTI is a rare, pigmented tumor that commonly occurs in craniofacial locations, and typically present as a localized pigmented swelling without pain or tenderness. Considering the rapid growth and that MNTI is easily misdiagnosed, this tumor deserves more attention.^[7]^ The lesions are discernible radiographically and expand into the surrounding bone, and can even encircle and displace teeth. On magnetic resonance imaging, the soft tissue lesions, present as hypointense masses on T1 and T2-weighted images. Histologically, the presence of 2 cell types is a typical feature of MNTI, consisting of larger epithelioid melanocytes and smaller primordial neurons (neuroblasts). Currently, many immunohistochemical markers have been identified in NMTI tissues. For instance, under normal circumstances, most cells express cytokeratin, HMB-45, and vimentin, while the expression of S100 is less common.^[9]^

A few tissues within the tumor express synaptophysin without chromogranin-A, which is a neuroendocrine marker. It has been noted that a small number of patients have elevated levels of vanilliylmandelic acid in their urine, which is consistent with other tumors of neuroectodermal origin, such as neuroblastoma.

At present, surgical resection is the primary therapy for MNTI, but the margin of resection remains controversial.^[10]^ Most specialists recommend that during surgery for MNTI a 5-mm margin of the resection should be used as an attempt to remove all of the involved tissue, thus preventing a recurrence. Others indicate that it is not necessary to extend the margin range because the remaining tumor tissue has self-healing abilities.^[11]^ In addition, other therapies can be used, including chemotherapy and radiotherapy. However, it is still unclear whether chemotherapy can prevent metastasis. Therefore, neoadjuvant chemotherapy is only used for tumors that involve nerves, which cannot be directly surgically resected.^[8,12]^ However, Rachidi et al^[13]^ reported the recurrence rate for MNTI to be 15% to 27%, for which the reasons are multifaceted, including incomplete resection and the occurrence of multicentric tumors. The case presented here was an infant less than 1-year-old. She was admitted to the hospital with a left maxillofacial mass. The radiological and pathological characteristics of the mass were consistent with previously reported cases of MNTI. After complete resection, the tumor reoccurred 3 months later. Due to the infants inability to tolerate a second surgery, and the uncertain effectiveness of radiotherapy and chemotherapy, we decided to prescribe metformin for the infant. After 9 months of medication, the tumor had not progressed, and the infant was growing well, which indicated that metformin may have been unexpectedly effective in halting the growth of the MNTI.

Metformin is one of the most commonly used drugs for the treatment of type 2 diabetes mellitus. Interestingly, the prevalence of tumors in patients with diabetes taking metformin is significantly lower than in patients using other hypoglycemic agents.^[14]^ A large number of interventional and molecular studies have demonstrated that metformin has anti-tumor properties,^[11,15,16]^ but the possible mechanisms underlying the properties remain unknown. In the past, metformin has been proven to inhibit GLS (glutaminase) activity and ammonia accumulation,^[17]^ thereby reducing the risk of some diseases in patients with type 2 diabetes mellitus. Since tumor cells are highly glutamine dependent and overexpress glutaminase, the mechanism of tumor suppression of metformin could be due to inhibition of GLS and the reduction of ammonia and ammonia-induced autophagy.^[18]^ Furthermore, Serena et al have demonstrated that, consistent with this pathway, metformin targets mitochondrial GLS, which is involved in glutamine metabolism. Metformin also inhibits the α-ketoglutarate to tricarboxylic acid cycle, which leads to a deregulation of tumor cell metabolism.^[19]^ Metformin inhibits GLS, which reduces cellular ammonia accumulation that impairs autophagy.

It has been confirmed that the excessive activation of the mTOR (mammalian target of rapamycin) signaling pathway is closely related to cell metabolism, growth, and tumor proliferation.^[20]^ Recent research indicates that metformin can inhibit tumor growth by affecting the mTOR pathway. mTORC1 is one of the complexes that contain the catalytic subunit, mTOR, which is essential for cellular proliferation and energy metabolism. The mTORC1 complex can receive different signal pathways, such as IGF1 (insulin-like growth factor 1) and IGF2 via AMPK (AMP-activated protein kinase).

Typically, metformin inhibits the mTORC1 complex through the phosphorylation of RAPTOR (regulatory-associated protein of mTOR) via AMPK.^[21]^ Metformin also blocks the tumor suppressor genes, TSC1, and TSC2, through the IGF1 and insulin signaling pathway, thereby exerting an inhibitory effect on mTORC1. The tumor suppressor protein, p53, which is induced by metformin, can activate AMPK, which leads to mTORC1 inhibition and suppression of tumor cell proliferation.^[18]^ However, additional potential mechanisms remain ambiguous. Undoubtedly, determining which specific signal pathway or pathways metformin affects MNTI needs further study.

Because MNTI is a rare tumor with a relatively high recurrence rate, the current strategies for treatment are too simple, and the development of novel therapies is slow. This case report provides a potential new direction to treat recurring MNTI clinically. This report also should inspire clinicians to consider novels uses of currently used drugs to treat rare diseases especially tumors.

## 4 Conclusion

MNTI is an extremely rare disease, and the available therapies are underdeveloped. Based on this case report, metformin is an easily accessible drug that has the potential to become an ancillary treatment for MNTI, as well as other rare diseases. However, our sample was small. Therefore, additional, randomized controlled trials with larger samples are needed to confirm our observation.

## Acknowledgments

We thank Dr. Song Gu for sharing he expertise in treating patients with MNTI and his selfless help. All authors have contributed to the manuscript in significant ways, have reviewed and agreed upon the manuscript content. And we are grateful to Professor Ji Tong, Director of Oral and Maxillofacial Surgery, Shanghai Ninth People's Hospital, Shanghai Jiao Tong University School of Medicine, for his assistance in the operation. We thank Editsprings (www.edit.springs.com) for its linguistic assistance during the preparation of this manuscript.

## Author contributions

Yu Liang proposed this idea and completed most of writing.

Ruicheng Tian Participated in literature searching and writing.

Jing Wang, Yuhua Shan, Hongxiang Gao, Chenjie Xie, Jingjing Li, Lei Zhang and spent a lot time screening and analyzing literatures.

Min Xu and Song Gu contributed the case and provided financial support.

**Conceptualization:** Yu Liang, Lei Zhang.

**Data curation:** Yu Liang.

**Formal analysis:** Yu Liang, Ruicheng Tian, Yuhua Shan.

**Funding acquisition:** Song Gu.

**Resources:** Jing Wang, Yuhua Shan, Hongxiang Gao, Chenjie Xie, Jingjing Li, Lei Zhang, Min Xu.

**Supervision:** Jing Wang, Yuhua Shan, Hongxiang Gao, Chenjie Xie, Jingjing Li, Lei Zhang, Min Xu, Song Gu.

**Writing – original draft:** Yu Liang, Ruicheng Tian.

**Writing – review & editing:** Yu Liang.
